# Fatal aluminum phosphide poisonings in Tirana (Albania), 2009 – 2013

**DOI:** 10.1186/s40199-015-0090-0

**Published:** 2015-01-25

**Authors:** Zihni Sulaj, Alert Drishti, Irena Çeko, Amarda Gashi, Gentian Vyshka

**Affiliations:** Service of Clinical Toxicology and Addictology, University Hospital Center “Mother Theresa”, Tirana, Albania; Biomedical and Experimental Department, Faculty of Medicine, University of Medicine, in Tirana, Albania

**Keywords:** Poisoning, Aluminum phosphide (AIP), Pesticides, Mortality, Phostoxin

## Abstract

**Background:**

Acute poisonings particularly through pesticides have become a major public health concern in Albania during the last decade.

**Findings:**

The number of fatalities due to aluminum phosphide intoxications was more than doubled during a five year-period from 2009 to 2013, and a cluster of suicides perpetrated with Phostoxin was registered. Several factors are accountable for such a phenomenon, including the fact that aluminum phosphide agents are freely available in the Albanian market, their price is extremely low and they are sold without any legal restriction. The mass media unfortunately warranted an emulating effect to dramatic intoxications, which gained by such means the notoriety of a secure lethal weapon.

**Conclusions:**

Our experience with more than three hundred intoxications with aluminum phosphide agents in the last five years, showed that a considerable delay from the moment of exposure (mainly through ingestion) to specialized medical help seeking, created a considerable obstacle for a successful treatment of cases, and eventually for the survival of patients. The lack of a specific antidote adds further challenges to all these exposures. The need for public health policies aiming at prevention, awareness, and possibly the substitution of Phostoxin or other aluminum phosphide pesticides with less dangerous agents is formulated.

## Background

Acute pesticide poisonings of a deliberate or accidental nature are a major public health issue in almost every country [1-3]. Studies and statistical data related to Albania are scarce, fragmented or even inexistent. However, recent publications have raised concerns regarding a constant increasing trend of suicide rates in Albania, with self-poisoning being a method of choice [4]. The same seems to be valid for attempted suicides, with more than 97% of adolescents trying to self-poison themselves with pharmaceuticals [5].

Agrochemical poisoning and related deaths, particularly AIP intoxications and fatalities, have gradually become a major public health concern for the Albanian society during the two last decennia. This is not strictly an Albanian phenomenon, since the toxicological situation is problematic in many countries, with the increased use of chemicals in the agriculture being a major causative factor [6].

Self poisoning through pesticides accounts for approximately 20 to 25% of all suicide in Western Mediterranean, Africa and South-East Asia, whereas half of suicides in Western Pacific were related to pesticides [7,8]. If we consider global data, pesticides should be accountable for 350.000 to 440.000 deaths every year, with 99% of those poisonings occurring in developing countries [9].

Poisonings with AIP have recently become a major public health concern in Albania. In addition to accidental poisonings, a large number of suicides have been registered, followed from homicidal fatalities and unclear cases, when the intoxication albeit not accidental, was seemingly purposeless. With dozens of cases reported in the local press, and presumable much more taciturnly not reported, journalists have anticipated the ominous omnipresence of AIP inside Albanian markets. In a recently published article, “Suicide is worth of an aspirin”, the media underlined the horrible fact that AIP, in the form of phostoxin, are freely available in the market, sold without any restriction, and with a single tablet costing 25 Albanian leks (local currency), equaling with a quarter US dollar [10].

**Figure 1:**
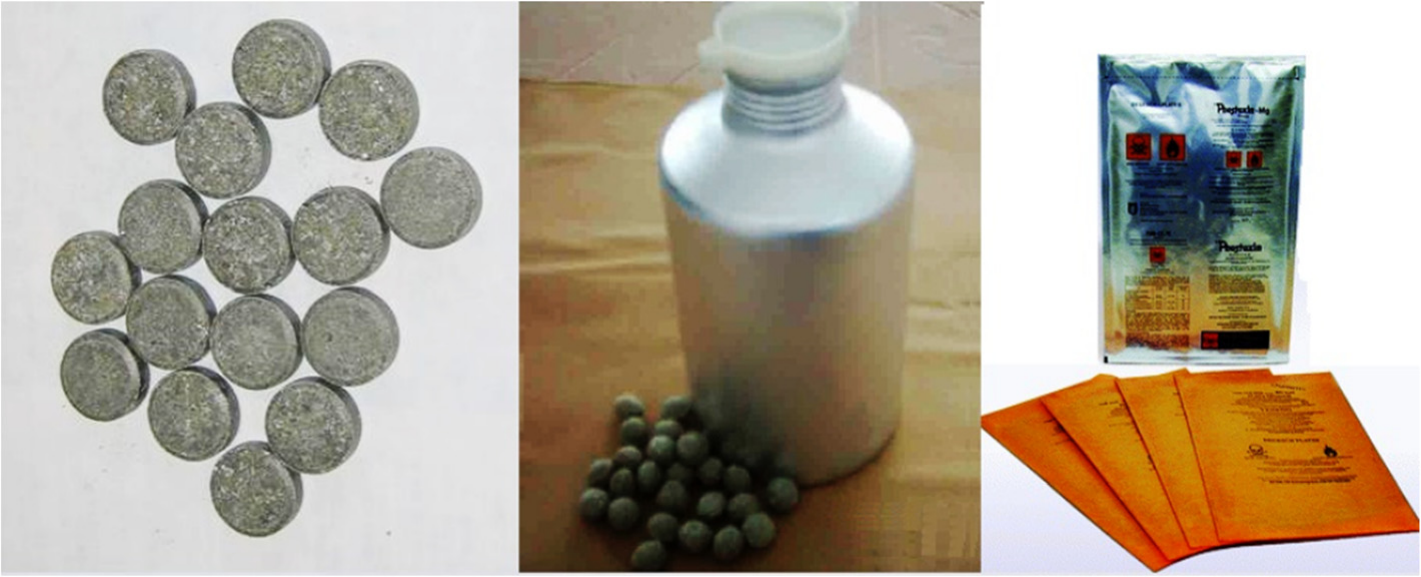
**Right insert; dark-brown tablets of Phostoxin.** Middle and left insert; other forms of the same product freely sold in the Albanian market.

Aluminum phosphide (AIP) and aluminum carbonate in a ratio of 56:44 are the constituents of the product freely sold in Albania, mainly under the trade mark of Phostoxin, but with the name of Celphos as well. The product is usually marketed in metallic containers of ten tablets, each of them weighting three grams. One tablet releases one gram of Phosphine (PH3), responsible for the toxic effects. The product is sold as pellets as well [Figure 1] weighting 0, 2 grams.

## Methodology

The present study was conducted in a single center, with an analytic retrospective collection of data. The center was the Service of Clinical Toxicology, University Hospital Center “Mother Theresa” of Tirana, capital of Albania. The suburban area and the downtown of Tirana actually count approximately one million inhabitants, equaling one third of the entire country population. However, being the only University center in Albania, the majority of severe intoxications are referred here. Therefore we might presume, with an acceptable margin of error, that the study population represents the trend of the entire country, with regard to Phostoxin intoxications and suicides.

Data collected has covered the period from January 1st 2009 to December 31st, 2013. The five-year period registered a total of 317 AIP intoxications, from which 140 were fatal cases. Diagnoses were picked up from patients’ charts; 40% of the cases were followed from forensic investigations and judicial autopsies. Such data were out of our reach, and the forensic aspects of the intoxications remained out of the scope of the present paper. Data were anonymously treated and the study was performed in compliance with the Helsinki Declaration. The approval of the Ethical Committee was requested and granted as well.

Our service is specialized for non-pediatric patients, so only victims aging more than 14 years are admitted and treated herein. Therefore we do not possess as well data regarding pediatric intoxications.

*Inclusion criteria* for the patients recruited in the study were: clinical picture suggestive of AIP intoxication and history of the event (as referred from relatives or patient, when possible); laboratory tests including gas-chromatography analyses were applied in suspicious cases. *Exclusion criteria* for the present study were situations when patients were declared dead on arrival, pediatric ages (already treated in another facility and out of our scope), and for intoxications without a typical clinical syndrome and history for an AIP consumption.

## Findings

From an overall number of 3656 admissions during the five year period covered from the study (2009–2013) we had 317 poisoned with aluminum phosphide products. Thorough data were retrospectively collected from the files of 140 fatalities. A summary of the demographic characteristics is presented at the Table 1.

**Table 1:** **Demographic data of the study group (140 cases with fatal AIP intoxication)**

***Parameter***	**Sex**	**Geographic origin**	**Mean age**	**Mean age, female subgroup**	**Mean age, male subgroup**	**Case/ratio fatality**
	81 females (58%)	76 from urban areas (54%)	35 years (min. 16 years; max. 71 years)	30 years (min. 16 years; max. 69 years)	39 years (min. 17 years; max. 71 years)	317/140 (44%)
	59 males (42%)	64 from rural areas (46%)				

The *diagnosis* of AIP was made based on the history, the clinical symptoms and the characteristic smell of rotten fish of gastric contents. The rapid diagnosis test with silver nitrate (AgNO_3_) was regularly performed when gastric content was collected from vomited material. Silver nitrate solution was as well used to test the patient’s breath through a moistened paper; its blackening is strongly suggestive of PH_3_ exhalation thus of an AIP intoxication [11]. Gas Chromatography with head-space Gs chromatograph analysis (Shimadzu GC-2010 equipped with a Shimadzu AOC-201 autosampler system, Japan) was performed in 68 of the fatalities, when other tests resulted negative.

**Figure 2:**
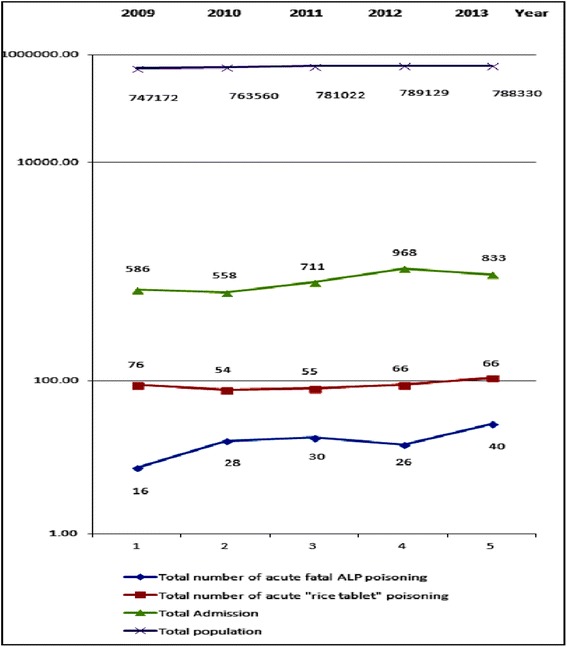
**Schematic drawing of the data summarized in the Table** 2 **will suggest an increasing number of fatalities, in an otherwise stable total population of the city in study (Tirana), as well as of the totality of admissions due to AIP intoxications.**

There was a consistent increase in the time trend of fatal intoxications, with values being gradually but constantly higher from year to year, 2012 exclusive (see Table 2 and Figure 2 below).

**Table 2:** **Time profile of total and fatal intoxications, the annual incidence of fatalities and the population under study (Tirana, 2009–2013)**

**Year**	**Total number of acute fatal AIP poisonings**	**Total number of acute AIP poisonings**	**Total admissions**	**Annual incidence of fatal AIP poisonings**	**Total population of Tirana (capital of Albania, official sources)**
**2009**	16	76	586	0.21	747172
**2010**	28	54	558	0.52	763560
**2011**	30	55	711	0.55	781022
**2012**	26	66	968	0.39	789129
**2013**	40	66	833	0.61	788330
**Total number**	**140**	**317**	**3656**

**Figure 3:**
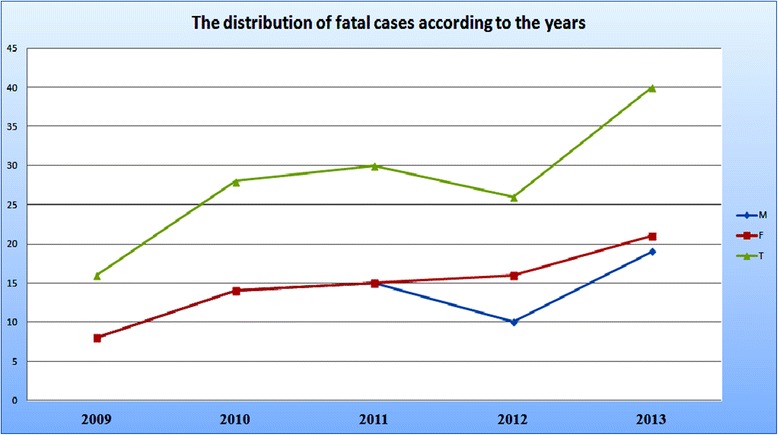
**Distribution of fatalities during the years of the study, and the gender differences (M – males; F- females, T-Total) that have become visible only during 2012 and 2013, with a female preponderance.**

Apart from the suggestive finding of an increasing incidence of fatal intoxications during the years (0, 21 in 2009 vs. 0, 61 in 2013), important details could be picked up by studying the age profile, and sex, of the intoxicated patients. Our focus was obviously on the fatal cases, and we had a total of 22 deaths from the 16–19 years age subgroup that represented 15, 7% from the total of 140 fatalities, and in this precise subgroup the male–female ratio was 1:5, 6, whereas such a ratio was 1: 1, 5 in the total group. Differences were seen as well in the yearly distribution of this ratio for the total group, but only during the last two years of the study (See Table 3 and Figure 3 below).

**Table 3:** **Age profile of fatalities during the study time period (Tirana, 2009–2013)**

**Age group**	***2009***	***2010***	***2011***	***2012***	***2013***
**Unidentified**				2	3
**16-19 years**	3	3	5	3	8
**20-25 years**	1		5	9	4
**26-29 years**	1	1	1	2	1
**30-35 years**	1	4	5	5	5
**36-39 years**		1		1	2
**40-45 years**	2	4	3	2	6
**46-49 years**	3	2	3		3
**50-55 years**	3	5	4		2
**56-59 years**	1	1			
**>60 years**	1	7	4	2	6
**Total**	16	28	30	26	40

The *main route of exposure* with the toxic product was through ingestion (96% of cases); only a small minority lacked data. Out of 140 fatalities we had only two cases poisoned with zinc phosphide; all other victims were intoxicated with aluminum phosphide. Concerning the *circumstances* of the poisoning, the occurrence was suicidal in 132 cases (94%); in 6% of cases no obvious suicidal intention could be confirmed, beyond reasonable doubts.

An important variable with regard to the outcome of AIP intoxications was the time from exposure to treatment initiation. Despite obvious difficulties in calculating a precise moment of exposure, due to variety of reasons (victim unconscious/hesitating relatives/unclear picture of intoxication etc.) we had to deal with cases admitted in the hospital from a minimum of 30 minutes after intoxication, to a maximum time delay surpassing 24 hours. The mean period of *time from exposure to hospital admission* was calculated 3, 91 hours (±5, 11 hours).

The dose of AIP product was as well not reliably evidenced in the medical files, since data were collected from relatives’ confessions, when reliable. The dose of AIP varied from half a tablet (one tablet weighting 3 grams) to five tablets (meaning a dose of the active component [i.e. phosphine] from 0, 5 to 5 grams).

The clinical status of the victims was also variable upon hospital admission; 52 cases were already in deep coma on arrival (Glasgow coma scale 3–6 points); symptoms and signs and respective frequencies are summarized in the Table 4.

**Table 4:** **Symptoms, signs of the clinical picture and laboratory findings upon arrival (140 fatalities)**

***Symptom/sign/laboratory finding***	***No. of patients encountered***	***Frequency***
Cardiovascular collapse	128	92%
Profound thirst	74	53%
Nausea	67	48%
Vomiting	65	46%
Abdominal pain	60	43%
Chest pain	55	39%
Coma (GCS less than 7 points)	52	37%
Cyanosis	42	30%
Restlessness	40	29%
Pulmonary edema	20	14%
*Metabolic acidosis*	123	88%
*Hypomagnesemia*	64	46%
*Hyperglycemia*	22	16%
*Sinus tachycardia*	61	44%
*Other cardiac rhythm disorders*	15	11%

Although the treatment of AIP intoxications is still disappointing and non standardized, different therapeutic options are suggested and are as well available in our center. We carried out a gastric lavage with potassium permanganate (1:10000) in 68 of patients. Taking into account the relative long mean period of time separating the exposure from the arrival in hospital (averaging four hours), the gastric decontamination would have a questionable role, if any. Supportive care, hemodynamic monitoring, oxygenation, fluid and electrolyte substitution, were all offered during the hospitalization. Mechanical ventilation was applied in 46 patients out of 140 fatalities, obviously without influencing the lethal outcome of the occurrence.

The hospital length of stay (LOS) was an average of 3, 5 days; with 85% of the victims passing away during the first twenty-four hours. The case-fatality ratio (CFR) in this series was 44% (140 deaths in a total of 317 AIP intoxications); CFR was not constant year after year. Unfortunately and in spite of an increased experience gathered in the field, we had a higher CFR during 2013, with 61% of AIP intoxications having a fatal outcome. With this figure being an isolated finding, such a fact is still debatable.

## Discussion

Phostoxin is a highly effective insecticide and rodenticide, used for in-door and out-door environments. The fatal dose of this product is considered to be from a minimum of 150 milligrams to a maximum of 500 milligrams [11]. Literature suggests alarming figures of mortality rate following AIP poisoning, ranging from 40% to 80% [11,12]. Phosphine, the active component, inhibits the mitochondrial cytochrome oxidase and the cell oxygen utilization, disrupting at the end the normal mitochondrial activity [12,13].

Phostoxin is freely available in the Albanian market, imported from several countries, in first line from Turkey, India and China. It is widely applied as fumigant, insecticide, rodenticide and raticide. Due to the fact that Albania is not a producer of rice, the term ‘rice tablet’ might be erroneous for this context. Instead, phostoxin is used mainly in grain and beans handling facilities and in general for preserving cereals, a method which is used in other countries as well [14].

Aluminum Phosphide products have gained during the last decade the notoriety of agents that might safely cause someone’s death. This high level of lethality with sustained dosages (one tablet surpasses the potential for killing an individual), the overall availability, an extremely low price on the market and above all, an almost complete lack of safety measures, have caused a cluster of suicides during the last years in Albania. A big deal in this trend should have played the mass media, with the emulating factor intrinsic to publishing sensational notes of suicides, even when those were unverified. The mass media influence, although controversial and of unease acceptance, has been formulated from other authors as well [15]. Actually the events of suicides with phostoxin are as a rule printed on the cover page of Albanian daily newspapers.

The data collected from the files suggested some worth mentioning particularities. According to our study, and referring to other sources as well, the victims mainly belong to the third or fourth decade of life; with women attempting suicides in a younger age. We had a mean age of 35 years for the entire study group, with males aging in average on decade older than females, when victimizing themselves with AIP. A higher resolution picture on our data offered furthermore another particularity, when evaluating the male–female ratio. Thus, the 16–19 years age subgroup presented 15, 7% from the total of 140 fatalities, and in this precise subgroup the male–female ratio was 1:5, 6. This is a major change from the male–female ratio in the total group of study, where such a figure was 1:1, 5. This subgroup of female adolescents (aging 16–19 years old) is obviously at a higher risk; although larger studies might be needed to generalize findings.

Our data in regard of the age of victims was not very different from other sources. Khodabandeh et al. suggests other figures, with a male–female ratio in favor of men (55:45) and with mean age of patients slightly younger from our group of study, with his patients aging 26 ± 8, 9 years [16]. A Greek study group offers an age profile much closer to ours, with a mean age of males of 37 years, and females of 34 years; but these data were related to poisoning in general, and not strictly to AIP consumption [17]. There is, however, a generally accepted opinion that characteristics of victims differ substantially when comparing developed countries with developing nations [18]. In fact, authors from India and Iran have reported the most numerous and systematic studies regarding AIP poisonings, but African sources (Nigeria, Morocco) are available as well, with striking similarities in the age profile of affected groups, and suicidal intentions [14,19]. Instead the European authors, with few exceptions, restrict their findings in isolated case reports [20]. A German study group however, has collected and published data from 188 intoxications during a twenty year period in the Poison Center of Mainz, with only 28% of cases having suicidal purposes [21].

Suicidal act is a complex phenomenon, largely influenced by access to lethal means, cultural and social factors, particularly gender and personality traits, such as impulsivity [22]. Among the three main factors impacting the mortality of pesticide poisoning authors suggest the host (with age, gender and genetics), the toxic agent and the environment [23]. Interventions aiming at limiting the toxicity (through using less dangerous preparations) or restricting the accessibility of the pesticides are more feasible and fruitful [23]. Of course, the high level of in-door use of pesticides and their depositing close to houses will increase the risk for acute poisoning, particularly in rural areas of developing countries [24].

AIP products are highly toxic agents, albeit the incidence of mortality is quite variable, from 30% to 100% when 500 mg or more of AIP have been ingested [25]. Several factors influence substantially the final outcome, since death or survival is connected with the early vomiting of the product, the intensity of vomiting, the mixture of AIP product into water before ingestion, or its disintegration and loss of effect. In our study group we dealt with 140 fatalities out of 317 AIP intoxications, with a death ratio of 44%; slight gender differences were detected hereby as well. In fact, male patients had a fatal ratio of 50% when compared with 40% in female patients. This was straightforwardly related with the fact that men consumed larger quantities of AIP, leading to higher blood levels of phosphide, and a more severe intoxicating event.

The lack of a specific antidote is a major concern when treating AIP intoxications. Of course, several other (mainly supportive) therapeutic options might be tried, including gastric washing with sodium bicarbonate (alternatively the potassium permanganate), hydration, oral administration of charcoal, hyperbaric oxygen, coconut oil and other suggestions of unproven efficacy [26-28]. Intravenous magnesium sulphates, treatment of acidosis, fluid resuscitation, vasopresors, digoxin and trimetazidine have been tried as well, particularly when cardiogenic shock dominates the clinical picture [25].

Among the negative prognostic factors related with the fatalities reported from our study group, we consider the relatively high consumed dosage of the toxic agent (a mean of 1, 64 tablets weighting three grams each); the long period of time elapsed from the toxic exposure to the onset of treatment (a mean of approx. four hours); the lack of vomiting after product ingestion, and the depth of coma upon presentation. Other factors might be accountable for the severity and the outcome of the intoxication, with authors detailing several of those [29,30]. Ingestion of exposed or expired tablets, or usage of botanical tablets that are practically non-toxic, might be among the reasons of survival among intoxicated persons [31].

Our study has covered only a five-year period, and it was performed in one single center, namely in Tirana. Albeit this center is a University facility, it clearly does not cover the entirety of the Albanian territory, with such a fact limiting the overall validity of our data. Another limitation is the retrospective collection of findings from medical files, when omissions of facts are a notorious reality, especially when relatives try to conceal or disguise the suicidal intents of the patient, as they might do for a diversity of identified reasons [32,33]. The inaccessibility of forensic findings (autopsy data included), albeit generally out of our scope, might be another limitation of this study, which was conceived as a descriptive one.

## Conclusion

The case series presented here is certainly only a portion of a larger number of AIP fatal intoxications happening in Albania during the last decade. With the list of fatalities prolonging year after year, the problem seems out of the focus of health and social policies. The reasons why this product has overtaken all other forms of deliberate poisoning are complex and unstudied. Nevertheless, we consider that among these reasons should be included the easy and unrestricted access to the toxic agents, the lack of regulations, the inefficient legislation; as well as the notoriety of a ‘secure’ lethal product causing an almost certain and non-traumatic demise.

The AIP intoxications are a public health concern in developing countries, with some sources suggesting an improvement in the overall outcome, with more survivors during recent years [34]. This is however, obscured from the fact that there is still no antidote, which warrants enough severity and unclear if not infaust prognosis to each case intoxicated with aluminum phosphide. In our study group we had also a very high level (94%) of cases clearly intoxicated for suicidal purposes, with a predominance of female victims originating from rural areas of Albania, but with a higher mortality among male patients. Fatal cases were more than doubled from 2009 (16 cases) to 2013 (40 fatalities). Sharp increases in these poisonings have been reported in other countries as well [35].

These occurrences have led authors to suggest the total withdrawal of AIP from the markets, with the introduction of safer products [36]. Policies aiming at prevention of acute pesticide poisonings have been formulated as well, with the adaption of effective measures for a better education, a proper handling and more strict observation of these products [37,38]. Extending health services into communities at risk, such as rural areas, and the improvement of level of access to specialized care, with regard to management of victims of poisoning, are as well very important steps that need to be adopted in this field. This is even more important in our context, since we could not identify a trend of improved survival in the recent years, in spite of the considerable bulk of experience collected in the field.

## Consent

Written informed consent was obtained from the patients for the publication of this report and any accompanying images.
